# Direct Association of Unfolded Proteins with Mammalian ER Stress Sensor, IRE1β

**DOI:** 10.1371/journal.pone.0051290

**Published:** 2012-12-07

**Authors:** Daisuke Oikawa, Akira Kitamura, Masataka Kinjo, Takao Iwawaki

**Affiliations:** 1 Iwawaki lab, Advanced Scientific Research Leaders Development Unit, Gunma University, Maebashi, Gunma, Japan; 2 Research Fellow of the Japan Society for the Promotion of Science, Chiyoda-ku, Tokyo, Japan; 3 Iwawaki Initiative Research Unit, Advanced Science Institute, RIKEN, Wako, Saitama, Japan; 4 Laboratory of Molecular Cell Dynamics, Faculty of Advanced Life Science, Hokkaido University, Kita-ku, Sapporo, Japan; 5 PRESTO, Japan Science and Technology Agency, Kawaguchi, Saitama, Japan; Ecole Polytechnique Federale de Lausanne, Switzerland

## Abstract

IRE1, an ER-localized transmembrane protein, plays a central role in the unfolded protein response (UPR). IRE1 senses the accumulation of unfolded proteins in its luminal domain and transmits a signal to the cytosolic side through its kinase and RNase domains. Although the downstream pathways mediated by two mammalian IRE1s, IRE1α and IRE1β, are well documented, their luminal events have not been fully elucidated. In particular, there have been no reports on how IRE1β senses the unfolded proteins. In this study, we performed a comparative analysis to clarify the luminal event mediated by the mammalian IRE1s. Confocal fluorescent microscopy using GFP-fused IRE1s revealed that IRE1β clustered into discrete foci upon ER stress. Also, fluorescence correlation spectroscopy (FCS) analysis in living cells indicated that the size of the IRE1β complex is robustly increased upon ER stress. Moreover, unlike IRE1α, the luminal domain of IRE1β showed anti-aggregation activity *in vitro*, and IRE1β was coprecipitated with the model unfolded proteins in cells. Strikingly, association with BiP was drastically reduced in IRE1β, while IRE1α was associated with BiP and dissociated upon ER stress. This is the first report indicating that, differently from IRE1α, the luminal event mediated by IRE1β involves direct interaction with unfolded proteins rather than association/dissociation with BiP, implying an intrinsic diversity in the sensing mechanism of mammalian sensors.

## Introduction

The endoplasmic reticulum (ER) is responsible for the structural maturation of proteins entering the secretory pathway. To ensure the fidelity of protein folding and maturation, cells turn on a network of signaling pathways, collectively termed the unfolded protein response (UPR) [1]. Protein folding is monitored by three distinct sensors: inositol-requiring enzyme 1 (IRE1) [2]–[3]; protein kinase RNA (PKR)-like ER kinase (PERK) [4]; and activating transcription factor 6 (ATF6) [5]. IRE1 is a conserved transmembrane protein that has an ER luminal sensor domain and cytosolic kinase and ribonuclease domains. The luminal domain of IRE1 senses the accumulation of unfolded proteins and then the activated ribonuclease domain cleaves specific exon-intron sites in the mRNA encoding the transcription factor XBP1 (X-box binding protein 1) [6]–[7]. This cleavage initiates an unconventional splicing reaction, leading to the production of active XBP1 and induction of various adaptive genes [8].

The mechanism explaining how IRE1 senses the unfolded proteins is best understood in yeast. A series of studies identified the two-step sensing mechanism of yeast Ire1, consisting of a BiP-deprivation step and a direct association step. Under normal conditions, ER chaperone BiP is associated with Ire1. Under stressed conditions, excess unfolded proteins deprive Ire1 of BiP, and the resulting BiP-free Ire1 forms homomeric associations (Step 1) [9]–[12]. Then the homomeric Ire1 associates directly with unfolded proteins, which may elicit conformational change in the luminal domain, leading to the reorientation of the cytosolic domain and possible autophosphorylation of the kinase domain (Step 2) [13]–[14]. Moreover, recent studies indicate that the activated Ire1 clusters into discrete foci and forms dot-like assemblies [14]–[15]. Since the target RNA of yeast Ire1, Hac1 mRNA, is co-localized with these foci in cells, the high-ordered assemblies are believed to provide a concentrated, specialized molecular microenvironment, which could attract low-affinity binders with high avidity [16].

Similar to the yeast Ire1, mammalian IRE1α also senses the unfolded proteins via BiP dissociation and following homomeric association [17]–[20]. However, the mechanism is different from that of yeast Ire1. Compared to yeast Ire1 activation, which is dually regulated by BiP deprivation and direct association with unfolded proteins, the activation of mammalian IRE1α is mainly regulated by the BiP deprivation step [21]. It was recently reported that mammalian IRE1α also forms high-ordered assemblies upon ER stress [22].

In mammals, there are two IRE1 paralogues, IRE1α and IRE1β [23]–[25]. The major difference is their expression pattern. While IRE1α is expressed ubiquitously, the expression of IRE1β is restricted to the epithelium of the gastrointestinal tract [26]. Also, there is considerable divergence in their downstream events. Contrary to the survival effect mediated by IRE1α [27], IRE1β is involved in apoptotic cell death [25]. One reason behind this would be the diverse characteristics in the cytosolic domain, and the completely different targets: IRE1α cleaves XBP1 or insulin mRNA [28]–[30], while IRE1β targets ribosomal RNA [25] [31] or MTP mRNA [32].

Thus, even though the downstream effects mediated by the cytosolic domains of the two mammalian IRE1s have been extensively studied, their luminal events, especially in IRE1β, have not been elucidated. In this study, we performed a comparative analysis using the two mammalian IRE1s to clarify the luminal event mediated by IRE1β.

## Materials and Methods

### Plasmids

pTKbasal-h*IRE1*α-mEGFP-Flag (wild type, D123P, or K599A mutants) or pTKbasal-h*IRE1β*-mEGFP-Flag (wild type or K547A mutant) were used for the expression of GFP-fused human *IRE1s*. To make these plasmids, the PCR-amplified monomeric GFP (mEGFP) fragment, which contains A206K substitution, with 1x Flag tag was ligated into pTKbasal [21] using *Bam*HI/*Nhe*I sites. Then, the PCR-amplified human *IRE1*α fragment (stop codon removed) or human *IRE1β* fragment (stop codon removed) was inserted using *Hind*III/*Xho*I or *Hind*III/*Bam*HI sites, respectively. Mutations were introduced by PCR techniques. pCAG-h*IRE1α*-HA (wild type or K599A mutant) and pCAG-h*IRE1β*-HA (wild type or K547A mutant) were used for overexpression of 3x HA-tagged IRE1s [25]. For the overexpression of mouse *Amy*1, pCAX-m*Amy*1-Flag was used. To make this plasmid, PCR-amplified Amy1 fragment containing 3x Flag-tag was ligated into pCAX using *Hind*III/*Xho*I sites. For the overexpression of TCRα-GFP, pCAX-TCRα-GFP-Flag was used. To make this plasmid, PCR-amplified TCRα-GFP fragment containing 3x Flag-tag was ligated into pCAX using *Kpn*I/*Nhe*I sites. For bacterial expression of MBP-fused luminal fragments, pMAL-CSSR-His (for yeast Ire1) [33], pMAL-h*IRE1α*LD-His (for human IRE1α), and pMAL-h*IRE1β*LD-His (for human IRE1β) were used. To make pMAL-h*IRE1α*LD-His, PCR-amplified fragment encoding aa 31–443 of human *IRE1α* and 8x His-tag was digested using *Bgl*II/*Xho*I and ligated into *Bam*HI/*Sal*I-digested pMAL-c2x (NEB). To make pMAL-h*IRE1β*LD-His, PCR-amplified fragment encoding aa 35–432 of human *IRE1β* and 8x His-tag was ligated into *Bam*HI/*Sal*I-digested pMAL-c2x (NEB). As a XBP1-Luc reporter, modified ERAI reporter (pCAX-HA-2xXBP1DDBD(anATG)-LUC-F) [34] was used to detect the activation of IRE1α pathway. As an ATF4-Luc reporter, UMAI reporter (pCAX-mATF4(1–275)-Luc-F or pCAX-hATF4(1–285)-Luc-F) were used to detect the activation of PERK pathway. This is the gene for fusion with the upstream mRNA region (from the intrinsic first Met) of the mouse or human *ATF4*, and luciferase (GL3; Promega) [35]. To express the ER-localized luciferase, pTKX-ER-GL4 was used in Figure S1B [36].

### Cell Culture, Transfection, and Treatment

HeLa cells and HEK293T cells were cultured at 37°C in DMEM supplemented with 100 U/ml penicillin, 100 µg/ml streptomycin, and 10% fetal bovine serum, in an atmosphere containing 5% CO_2_. Effectene® Reagent (QIAGEN) was used to introduce plasmid DNA into HeLa cells, and the calcium-phosphate-DNA precipitation method was used for HEK293T cells. To induce ER stress, cells were treated with tunicamycin (2.5 µg/ml) or thapsigargin (1 µM). The assays with IRE1−/− MEFs (Fig. S1A) were performed as described previously [21].

### Live Cell Imaging

Before imaging, cells were washed twice with HBSS, and replaced in phenol red-free DMEM (Invitrogen) supplemented with 25 mM Hepes-NaOH (pH 7.4) and 10% FBS. Images were collected by LSM 510 META confocal microscope equipped with a C-Apochromat 40x/1.2NA UV-VIS-IR Korr. water immersion objective lens (Carl Zeiss).

### FCS Measurements

FCS measurements were performed with a ConfoCor 2 system and C-Apochromat 40x/1.2NA UV-VIS-IR Korr water immersion objective lens (Carl Zeiss) [37]. GFPs were excited at 488 nm. Confocal pinhole diameters were adjusted to 70 mm. Emission signals were detected with a 505 nm long-pass filter, and measured at 37°C in 5% CO_2_, 95% air-humidified atmosphere. The fluorescence autocorrelation function, *G*(t), from which the average residence time (t) and the absolute number of fluorescent proteins in the detection volume are calculated, was obtained as follows:
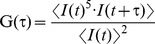
where *I*(t+t) is the fluorescence intensity obtained by the single photon counting method in a detection volume at a delay time t (brackets denote ensemble averages). Curve fitting for the multi-component model is given by:



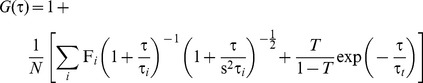
where *F*_i_ and t_i_ are the fraction and diffusion time of component *i*, respectively; *N* is the average number of fluorescent molecules in the detection volume defined by the beam waist *w*_0_ and the axial radius *z*_0_; s is the structural parameter representing the ratio of *w*_0_ and *z*_0_; *T* is the triplet fraction and t*_t_* is the relaxation time of the triplet state. In living cell analysis, a two-component model was used for the determination of diffusion time. *G*(t)s in living cells were measured for 30 s. The relationships between diffusion time and structural parameters were determined using a 10^−7^ M Rhodamine 6G (Rh6G) solution as a standard before measurement. The values of structural parameters were 4.5–6.0.

### Recombinant Protein Technique

Expression and purification of recombinant proteins were performed as described previously [14] [21] [33]. Escherichia coli strain BL21 CodonPlus™ (DE3)–RIL (Strategene) was used for the expression of each protein. Each MBP-fused protein was induced by 0.3 mM IPTG for 1 h at 37°C, and purified by Ni-NTA (QIAGEN). Each purified protein was subjected to SDS-PAGE (8% polyacrylamide) and CBB staining.

Anti-aggregation assays using purified recombinant proteins were performed as described in our previous reports [14] [21]. Citrate synthase (Roche) or luciferase (Promega) was dissolved at 70 µM in 20 mM HEPES (pH 7.2), 50 mM KCl, 2 mM MgCl_2_, 6 M Gdn-HCl. After denaturation for 1 h at room temperature, samples were diluted out of the denaturant to 1.5 µM in the case of citrate synthase or 1 µM for luciferase in 100 µl of 20 mM HEPES (pH 7.2), 50 mM KCl, 2 mM MgCl_2_, with or without 1.5 µM (CS) or 4 µM (Luc) of recombinant proteins. Turbidity of the sample mixtures was monitored by measuring absorbance at 320 nm and normalized against the maximum value of the buffer sample.

### Luciferase Assay

In the dual luciferase assay with the XBP1-Luc reporter, ATF4-Luc reporter, or ER-Luc, phRL-TK (Promega) was used as an internal control. HEK293T cells were seeded in 24-well plates, then transfected with plasmid DNA. At 24 h after transfection, cells were lysed for a luciferase assay. Reporter activity was measured using the dual luciferase assay system (Promega) and a luminometer (Berthold). The results are shown as means ± SEM from triplicate experiments. Each value is shown as a fold induction normalized to that of mock transfectant (for overexpression) or nontreatment (for drug treatment), the value of which was set at 1.0. In Figure S1B, the value was shown as a Relative activity (RLU).

### RT-PCR

Total RNA was prepared from cells using Isogen reagent (Nippon Gene). A SuperScript® First-Strand Synthesis System (Invitrogen) was used to synthesize the cDNA, according to the manufacturer's instructions. cDNA for XBP1 was amplified by 35 cycles of PCR using the following primers: human Xbp1 sense primer, 5′-AGAACCAGGAGTTAAGACAGC-3′; human Xbp1 antisense primer, 5′-AGTCAATACCGCCAGAATCC-3′.

### Cell Lysis and Immunoprecipitation

For immunoprecipitation to detect the interaction with Amy1 or TCRα-GFP, cells were lysed with TNE buffer (10 mM Tris-HCl pH 7.5, 1% NP-40, 150 mM NaCl, 1 mM EDTA) containing a protease inhibitor cocktail (Sigma). For immunoprecipitation to detect the interaction with BiP, the cells were lysed in lysis buffer (20 mM HEPES, pH 7.5, 150 mM NaCl, 1 mM EDTA, 1% Triton-X, 1% glycerol) containing a protease inhibitor cocktail (Sigma) [21]. Anti-HA immunoprecipitation of the protein was performed with protein-G-conjugated sepharose beads (Protein G – Sepharose 4 Fast Flow; GE Healthcare) and anti-HA mAb 12CA5 (Roche) according to standard procedures.

The lysates and immunoprecipitates were denatured in SDS sample buffer (50 mM Tris-HCl, pH 6.8, 2% SDS, 50 mM DTT, 10% glycerol, and 1 µg/ml bromophenol blue). SDS-PAGE was performed to resolve the proteins in the lysate. After electrophoresis, the proteins were electrotransferred onto a polyvinylidene fluoride microporous membrane and immunodetection was performed using an anti-HA antibody (HA.11; Covance), anti-KDEL antibody (Stressgen) or anti-Flag antibody (Sigma) according to standard procedures.

## Results

### Clustering of IRE1α and IRE1β upon ER Stress

**Figure 1:**
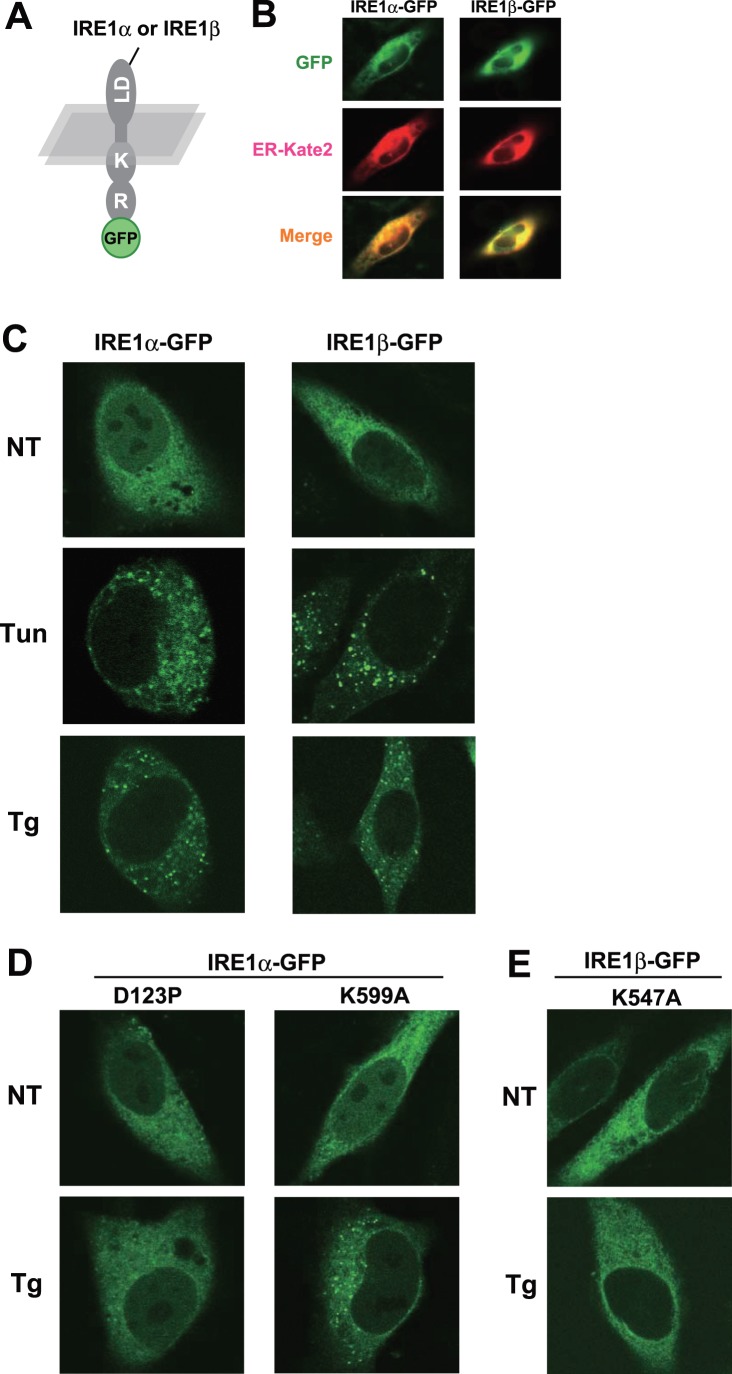
Clustering of IRE1α and IRE1β upon ER stress. (A) Schematic of IRE1-GFP imaging construct. Luminal domain (“LD”), kinase domain (“K”), RNase domain (“R”) of human IRE1s, and fused monomer-GFP are indicated. (B) ER-localization of GFP-fused IRE1s. Fluorescent images were collected from GFP-fused IRE1s and ER-mKate2 construct – transfected HeLa cells. (C) Clustering of IRE1α and IRE1β upon ER stress. GFP-fused IRE1s were transfected into HeLa cells and treated with or without tunicamycin (2.5 µg/ml for 2 h) or thapsigargin (1 µM for 2 h), after which fluorescent images were collected. (D, E) Clustering defect in mutant IRE1s. GFP-fused IRE1α (D123P or K599A) (D) or GFP-fused IRE1β (K547A) (E) were transfected into HeLa cells and treated with or without thapsigargin (1 µM for 2 h), after which fluorescent images were collected.

First, to elucidate the within-cell dynamics of the mammalian sensors, we fused monomeric green fluorescent protein tags (mEGFP) at the C terminus of IRE1s (Fig. 1A). As shown in Figure 1B, IRE1s were distributed on the ER, and could be merged with ER-localized markers (ER-mKate2). The functionality of GFP-fused IRE1s was confirmed with the induction of XBP1-Luc (IRE1α; Fig. S1A), and with the specific attenuation of ER-Luc (IRE1β; Fig. S1B). Under normal conditions, these GFP-fused IRE1s were diffusely distributed on the ER. However, upon ER stress, both IRE1α (Li et al., 2010) and IRE1β clustered into discrete foci (Fig. 1C).

Surprisingly, there was a slight time-lag in the clustering between IRE1α and IRE1β. While IREβ showed a distinct dot-like structure just 1 h after Tunicamycin (Tun) treatment, the IRE1α cluster began to appear 2 h after the treatment (Fig. S2). Moreover, the contributing domain to their clustering was different between IRE1α and IREβ. While IRE1α required its luminal function as the homomeric defective mutation (D123P), not kinase defective mutation (K599A), inhibited the clustering, IRE1β seemed to require its kinase activity because K547A (kinase defective) mutant did not shown any dot-like structures even under ER stressed conditions (Fig. 1D and 1E). These results imply that the molecular mechanism underlying the foci-formation is distinct in IRE1α and IREβ.

### FCS Analysis with IRE1α and IRE1β

**Figure 2:**
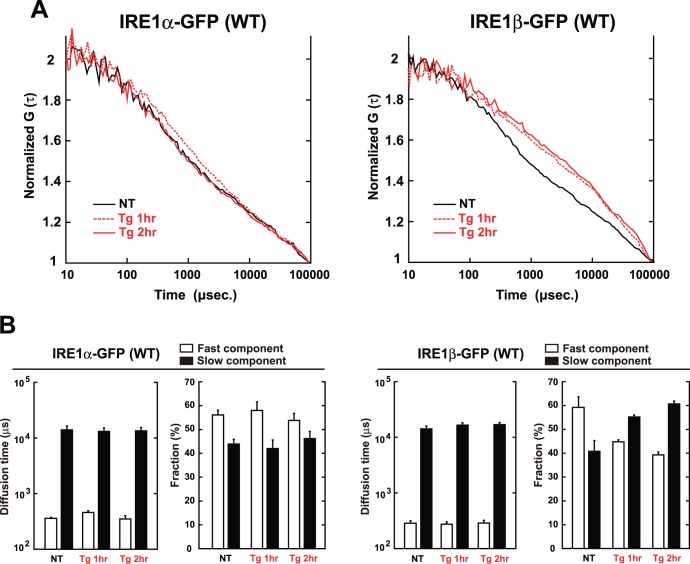
FCS analysis using IRE1α and IRE1β. (A) Normalized G(τ) of GFP-fused IRE1s with or without Tg treatment. GFP-fused IRE1s were transfected into HeLa cells and treated with or without thapsigargin (1 µM) for the indicated time, after which FCS measurement was performed. (B) Average diffusion time, diffusion coefficients and average fraction of each component of GFP-fused IRE1s. The diffusion time and fraction were calculated by curve fitting (two-component model), and their averaged values are indicated. The values of fast components are indicated as open bars, and the values of slow components are indicated as solid bars. Error bars are the measured mean ± SEM (n = 7 cells).

Next, to scrutinize the within-cell dynamics of IRE1s more precisely, we employed a dynamic imaging method, fluorescence correlation spectroscopy (FCS). FCS measures the fluorescent molecules within a confocal-detection volume at near single molecule sensitivity, and estimates their molecular number, or diffusion coefficient which reflects the size of the containing complex [38]. In this study, this technique was applied to the GFP-fused IRE1s in living cells (Fig. 2).

In FCS analysis, both of IRE1α and IRE1β showed fast movement, indicating that the IRE1s are dynamic on the ER membrane. Compared with the case of IRE1α, the autocorrelation curve of IRE1β significantly and robustly shifted to slower movement upon ER stress (Fig. 2A). Curve fitting analysis of this data (performed by a two-component diffusion model which is best fitted for IRE1s) revealed that the curve shift in IRE1β was caused by the increment in relative content of slow component rather than the decrement in the diffusion coefficient itself (Fig. 2B). Although it is not clear which IRE1 situation or condition is reflected by each component (fast or slow), this data absolutely indicates that compared to IRE1α, the size of IRE1β-complex is robustly enlarged upon ER stress. As shown in Figure S3, the FCS-detected enlargement of IRE1β-complex did not require kinase activity, because such shift was also detected in the K547A mutant.

What cause the stress-dependent shift of IRE1β? Generally, a smaller diffusion coefficient indicates that the diffusional mobility of a molecule is decreased. Indeed, the increase in the amount of IRE1β showing smaller diffusion coefficient suggested that the amount of IRE1β homomeric-association and/or binding with other proteins to make a huge complex (whose diffusion coefficient is ∼ 0.4 µm^2^/s) would be increased. To identify the IRE1β-associated factors that are not associated with IRE1α, a series of assays was performed as follows.

### Association of IRE1β with Model Unfolded Proteins

**Figure 3:**
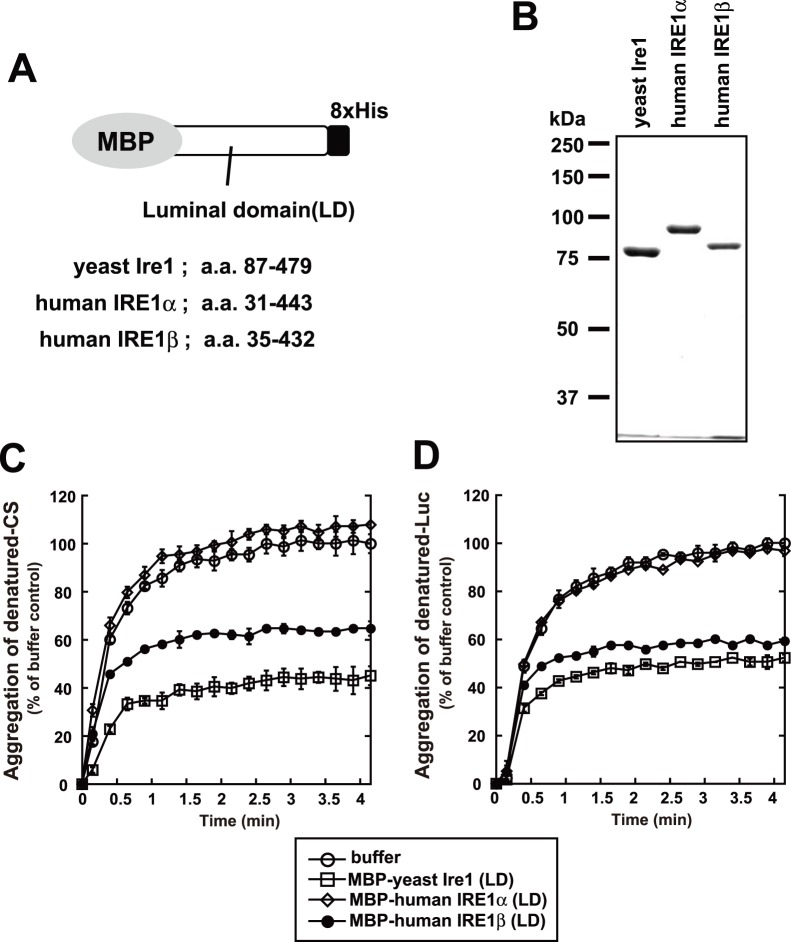
Anti-aggregation activity of IRE1β. (A) Schematic of recombinant fragments used in the anti-aggregation assay. (B) SDS-PAGE of recombinant fragments used in the anti-aggregation assay. Bacterially expressed fragments were purified by Ni-NTA, run on 8% SDS-PAGE gels, and stained with Coomassie blue. (C, D) Anti-aggregation assay with the recombinant fragments. At time 0, citrate synthase (C), luciferase (D) in guanidine HCl-denaturing buffer were diluted into assay buffer, with or without each recombinant fragment. Turbidity of the sample mixtures was monitored by measuring absorbance at 320 nm and normalized against the maximum value of the buffer sample. The average and SEM from three reactions are shown.

One hint came from the study of yeast Ire1. Yeast Ire1 has been shown to associate with unfolded proteins directly [14] [39], which may explain the IRE1s’ larger size-shift observed in the FCS analysis. To investigate this possibility for mammalian IRE1β, its luminal domain was prepared as MBP-fused fragments (Fig. 3A and 3B), and was subjected to *in vitro* anti-aggregation assay.

Strikingly, the luminal fragments of IRE1β exhibited robust anti-aggregation activity *in vitro*, by inhibiting the aggregation of denatured citrate synthase (Fig. 3C) or luciferase (Fig. 3D). Such activity was also detected in yeast Ire1, but not in mammalian IRE1α [21]. This indicates the possibility that, upon ER stress, IRE1β directly associates with unfolded proteins, and forms a larger complex.

**Figure 4:**
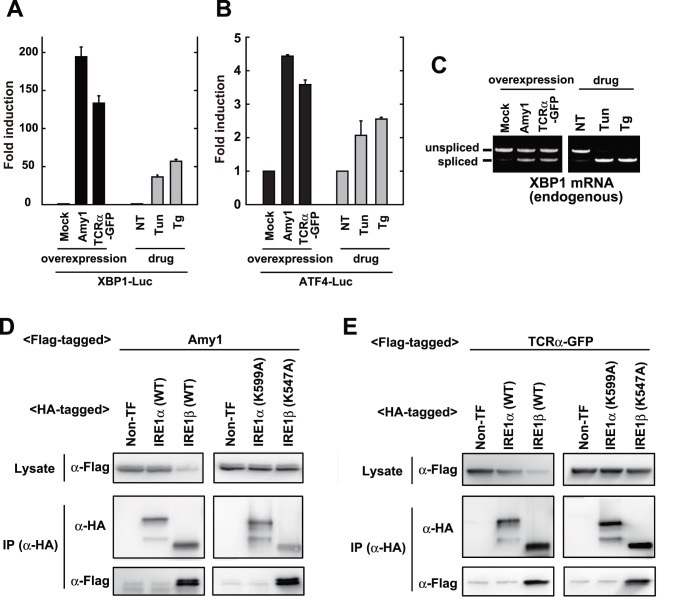
Association of IRE1β with model proteins. (A, B) UPR activation by the overexpression of model proteins. The XBP1-Luc reporter (A) or ATF4-Luc reporter (B) were co-transfected with or without Flag-tagged Amy1 overexpressing vector or Flag-tagged TCRα-GFP overexpressing vector into HEK293T cells. Luciferase assays were performed after treatment with or without tunicamycin (2.5 µg/ml for 8 h) or thapsigargin (1 µM for 8 h). (C) XBP1 splicing elicited by the overexpression of model proteins. Flag-tagged Amy1 overexpressing vector or Flag-tagged TCRα-GFP overexpressing vector was transfected into HEK293T cells. The cells were treated with or without tunicamycin (2.5 µg/ml for 2 h) or thapsigargin (1 µM for 1 h), and the total RNA was subject to RT-PCR. (D, E) Coprecipitation of IRE1β with model proteins. Flag-tagged Amy1 overexpressing vector (D) or Flag-tagged TCRα-GFP overexpressing vector (E) was co-transfected with or without HA-tagged IRE1 (wild type or mutants) overexpressing vectors into HEK293T cells. Their lysates were used for anti-HA immunoprecipitation. The cell lysates and the anti-HA immunoprecipitates were subjected to Western blot analysis.

Next, to evaluate the association of IRE1β with unfolded proteins in cells, immunoprecipitation was performed using Amy1 or TCRα-GFP as model substrates (Fig. 4). Previous reports indicated that the overexpression of Amy1 induces significant ER stress [40], and TCRα-GFP is known as an ERAD (ER-associated degradation) target [41]. As shown in Fig. 4A and 4B, the overexpression of these proteins caused considerable activation of UPR pathways, with the drastic activation of reporters including the XBP1-Luc (IRE1α-XBP1 branch; Fig. 4A), and ATF4-Luc (PERK-ATF4 branch; Fig. 4B). Also, the cytosolic splicing of XBP1 mRNA was induced by the overexpression of these proteins (Fig. 4C). The partially lowered splicing of XBP1 mRNA in Figure 4C (compared to Fig. 4A) would be attributed to transfection efficiency.

In the immunoprecipitation with these model unfolded proteins (Fig. 4D and 4E), the expression of Amy1 or TCRα-GFP was partially attenuated by the expression of IRE1β (wildype, not K547A mutant), due to the intrinsic RNase activity of IRE1β [36]. As expected, IRE1β showed strong coprecipitation signals, both with Amy1 (Fig. 4D) and TCRα-GFP (Fig. 4E), which was not detected in IRE1α. Also, since these associations were not inhibited by K547A mutation, kinase activity does not appear to be required for direct association with unfolded proteins.

### Different Pattern in the Association with BiP

**Figure 5:**
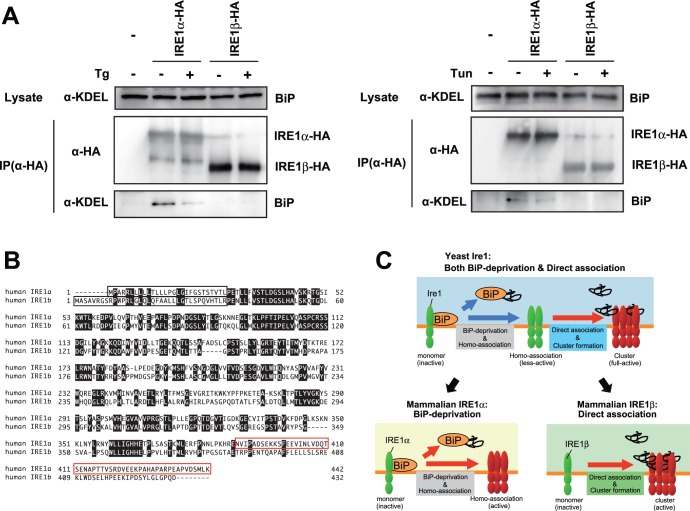
BiP-association with IRE1α, not with IRE1β. (A) BiP-association with IRE1α, not with IRE1β. HeLa cells transfected with the vector for overexpression of HA-tagged IRE1s (wild type or mutants) were treated with or without 2 µM thapsigargin for 30 min (left), or tunicamycin for 60 min (right), and their lysates were used for anti-HA immunoprecipitation. The cell lysates and the anti-HA immunoprecipitates were subjected to Western blot analysis. (B) Amino-acids sequence of luminal domain from IRE1α and IRE1β. Open black box indicated signal-sequences. Open red box indicated BiP-binding site on IRE1α. (C) Schematic representation of luminal events mediated by yeast Ire1, mammalian IRE1α, and mammalian IRE1β. See text for details.

Finally, we evaluated the association of IRE1β with ER chaperone BiP. IRE1α showed the BiP-association signal under normal conditions, and the signal weakened upon ER stress (Fig. 5A) [21]. However, and surprisingly, IRE1β did not show any BiP-association signal irrespective of ER stress (Fig. 5A). This implies the possibility that the luminal events mediated by IRE1β do not involve BiP association/dissociation.

## Discussion

In this study, we performed a comparative analysis using two mammalian IRE1s to clarify the luminal event mediated by IRE1β. One finding is that both IRE1α and IRE1β clustered into discrete foci upon ER stress, though the molecular mechanisms seem to be distinct in IRE1α and IRE1β (Fig. 1). FCS analysis indicated that IRE1β significantly and robustly shifts to a slower diffusion state upon ER stress, contrary to the IRE1α (Fig. 2). In agreement with this, the luminal domain of IRE1β showed anti-aggregation activity *in vitro* (Fig. 3), and IRE1β was coprecipitated with model unfolded proteins (Fig. 4). Another striking difference was found in the BiP-association pattern. While IRE1α was associated with BiP and dissociated upon ER stress as previously reported [17] [21], any association signals was not detected in IRE1β (Fig. 5A), which might be caused by their sequence differences on the region corresponding to the BiP-binding site of IRE1α (Fig. 5B) [21]. These results indicate that, differently from IRE1α, the luminal event mediated by IRE1β directly interacts with unfolded proteins. This study provides the significant information about the luminal event mediated by IRE1β, and also suggests the sensing mechanism of mammalian sensors may involve the specific pathway on signal transition during UPR.

To visualize the within-cell dynamics of IRE1s, we fused monomeric green fluorescent protein tags (mEGFP) at the C terminus of IRE1s. Monomeric substitution (A206K) may inhibit artificial oligomerization via intermolecular disulfide-bond between GFP. As previous studies [16] [22] inserted GFP-tag between the transmembrane and the cytosolic-effector domain, our C terminus adding of the GFP-tag might have eliminated the activity. However, this possibility was denied by the robust stress response of our GFP constructs (Fig. S1). Because all of these GFP-IRE1 (irrespective of the location of GFP-tag) showed stress-dependent clustering, it seems common activating mechanism to cluster into discrete foci rather than making small oligomer. However, the molecular mechanism behind the clustering seems different between the two IRE1 molecules, as IRE1β showed clearer and faster foci than IRE1α (Figs. 1 and S2; especially with Tun treatment), and as the kinase activity was only required for IRE1β not for IRE1α to clustered into discrete foci (Fig. 1).

Importantly, though primitive, this study contains trial experiment with FCS measurement of ER stress sensors. The only report evaluating the molecular diffusion of an UPR-involving factor is a FRAP (fluorescence recovery after photobleaching) analysis of the ER chaperone, BiP [42]. Molecular diffusion of BiP is decreased upon ER stress, which might be caused by the direct association with unfolded proteins to increase the size of the BiP-containing protein complex. Similarly, the molecular diffusion of IRE1β was decreased upon ER stress in our FCS analysis (Fig. S3), supporting the idea that IRE1β luminal events involve direct association with unfolded proteins under stressed conditions.

The clustering detected by fluorescent microscopy and the shift of autocorrelation curve in FCS analysis would reflect different molecular events, respectively, because FCS is detectable to only mobile molecules with a single molecule sensitivity [38]. Our results in Figure 1 showed that the cytosolic domain is important for the clustering of IRE1β. However, its shifts of autocorrelation curve did not need the cytosolic kinase activity (Fig. S3). Also, while both IRE1α and IRE1β clustered into foci upon ER stress, the robust shift to a slower diffusion was only detected in IRE1β (Figs. 1, 2 and S2). These observations would be implicated as two possibilities; (i) IRE1s (beta?) possess an ability to assemble without forming visible clusters, (ii) IRE1β is associated with unfolded proteins in the ER with a mobile state to rapidly recognize the substrates regardless the kinase activity in the cytosol.

The newly found differences between IRE1α and IRE1β at the activating step may involve the specific pathway on signal transition during UPR. It is thought that while IRE1α is transiently activated and attenuated soon to perform survival effect for cells [22] [27], the activation of IRE1β is continual to elicit apoptotic cell death [25], as implied from the sustained repression of MTP mRNA [43] and chronic change in intestinal lipid absorption [44]. Such difference in their activating timing or continuousness might contribute to their different downstream effects. Also, the substrates sensed by IRE1α and IRE1β might be different each other, resulting from their intrinsic sensing mechanism (Fig. 5C). The unfolded or malfolded status that elicits the deprivation BiP of IRE1α could differ from that directly binds to IRE1β, and the difference would determine which IRE1 molecule should be activated. As showon in Figure 5B, the amino acid-sequence between IRE1α and IRE1β is not so conserved on their luminal domain, especially in the adjacent region to the transmembrane domain that is the BiP-binding site in case of IRE1α [21]. This might be one reason behind the different activating step between IRE1α and IRE1β. Also, such selectivity might be one reason why the two sequential steps in yeast Ire1, the BiP-deprivation step and the direct association step, are evolutionally divided into each IRE1 molecules in mammal (Fig. 5C).

Still, some questions remain to be solved. One question is how IRE1β forms homo-associates or dot-like assemblies. Previous structure analysis reported that yeast Ire1 has multiple homomeric interfaces in its lumen and forms polymeric oligomers [45]. On the contrary, the luminal domain of mammalian IRE1α has a single interface and forms dimers or small oligomers [46]. At this time, we have no clear answer to whether the luminal domain of IRE1β forms dimers (small oligomers) or robust high-molecular oligomers, because there are no structural information about the luminal domain of IRE1β, and because the amino-acid sequence is not so conserved on their luminal domain (Fig. 5B). We could not estimate the oligomer size of the MBP-fused IRE1β (Fig. 2), due to several technical difficulties. Alternatively, the cytosolic domain which contains dimmer-forming interface in IRE1α [47] might contribute to the dot like assemblies also in IRE1β, as the cytosolic kinase activity was important for the clustering of IRE1β (Fig. 1). Another question is how the stress-dependent clustering of IRE1β is regulated. Although an association with BiP was hardly detected in our immunoprecipitation (Fig. 5A), we cannot exclude the possibility that BiP is involved in the clustering of IRE1β by a different manner than with IRE1α, as it has been reported that IRE1β is co-immunoprecipiated with BiP from extracts of mice stomach mucosa (a tissue rich in IRE1β) [26]. Alternatively, there may be other regulating factors. Recent reports on IRE1α describe various regulating factors associated with the cytosolic domain, including BI-1 [48] or RACK1 [49]–[50]. Such factors might exist to regulate the clustering or activation of IRE1β. Also, it is still unknown how the luminal events (clustering, or direct association with unfolded proteins) links to the cytosolic activation (or phosphorylation). Does direct association function as a regulating step for the activation of IRE1β? Moreover, we could not examine the activity of IRE1β in detail. Only a small number of targets specific to IRE1β have been reported, and a system for precise evaluation of their changes has not been developed. To overcome these problems, and to fully elucidate the sensing mechanism of IRE1β, further research is needed.

## Supporting Information

Figure S1 **Functionality of GFP-fused IRE1s.** (A) Functionality of GFP-fused IRE1α. The IRE1α expression vector (left) or IRE1α-GFP expression vector (right) were co-transfected with the XBP1-Luc reporter into IRE1α −/− MEFs. Luciferase assays were performed after treatment with or without tunicamycin (2.5 µg/ml for 8 h) or thapsigargin (1 µM for 8 h). (B) Functionality of GFP-fused IRE1β. The IRE1α-GFP (wildtype or K599A) expression vector, or IRE1β-GFP (wildtype or K547A) expression vector were co-transfected with the ER-luciferease reporter (left) or cytsolic-luciferase reporter (right) into HeLa cells, then luciferase assays were performed.
(PDF)

Figure S2 **Timing of cluster formation of IRE1α and IRE1β upon tunicamycin treatment.** GFP-fused IRE1s were transfected into HeLa cells, and treated with tunicamycin (2.5 µg/ml) for the indicated time. Fluorescent images were collected from untreated, 1-h treated, or 2-h treated cells.
(PDF)

Figure S3 **FCS analysis with mutants of IRE1α or IRE1β.** GFP-fused IRE1s were transfected into HeLa cells and treated with or without thapsigargin (1 µM) for the indicated time, after which FCS measurement was performed. Normalized G(τ) was shown.
(PDF)
