# Supplementary figures and images for: Direct Association of Unfolded Proteins with Mammalian ER Stress Sensor, IRE1β
Source: PLoS One. 2012 Dec 7;7(12):e51290. doi: 10.1371/journal.pone.0051290 (PMC3517461; doi:10.1371/journal.pone.0051290)

**A**

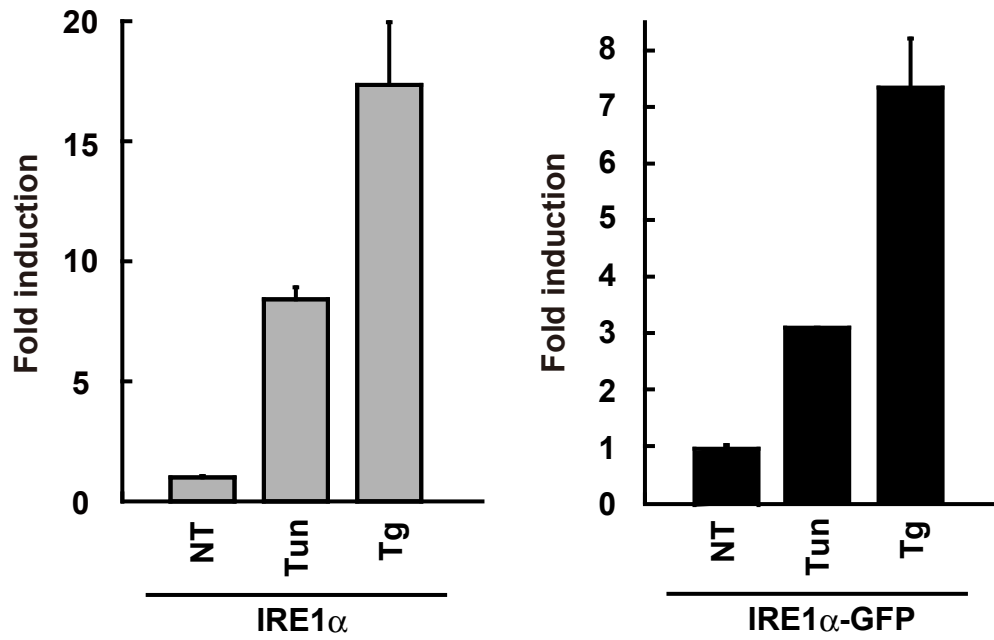

**B**

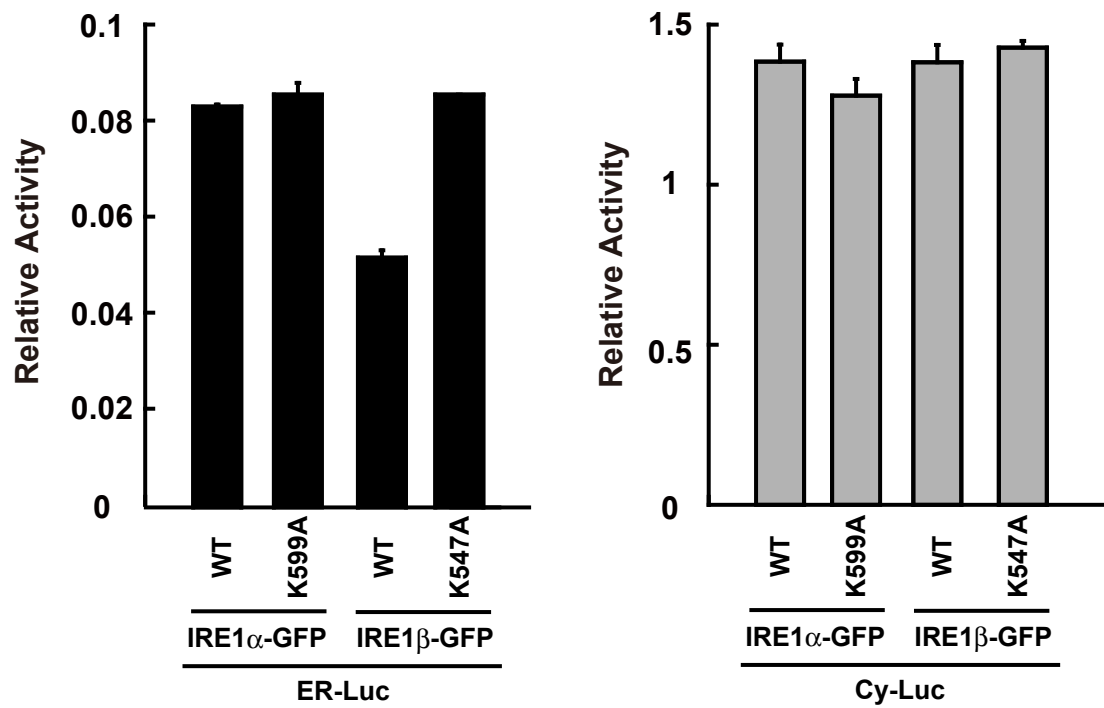

Supplement: Figure S1 — Functionality of GFP-fused IRE1s. (A) Functionality of GFP-fused IRE1α. The IRE1α expression vector (left) or IRE1α-GFP expression vector (right) were co-transfected with the XBP1-Luc reporter into IRE1α −/− MEFs. Luciferase assays were performed after treatment with or without tunicamycin (2.5 µg/ml for 8 h) or thapsigargin (1 µM for 8 h). (B) Functionality of GFP-fused IRE1β. The IRE1α-GFP (wildtype or K599A) expression vector, or IRE1β-GFP (wildtype or K547A) expression vector were co-transfected with the ER-luciferease reporter (left) or cytsolic-luciferase reporter (right) into HeLa cells, then luciferase assays were performed. (PDF) [file pone.0051290.s001.pdf]

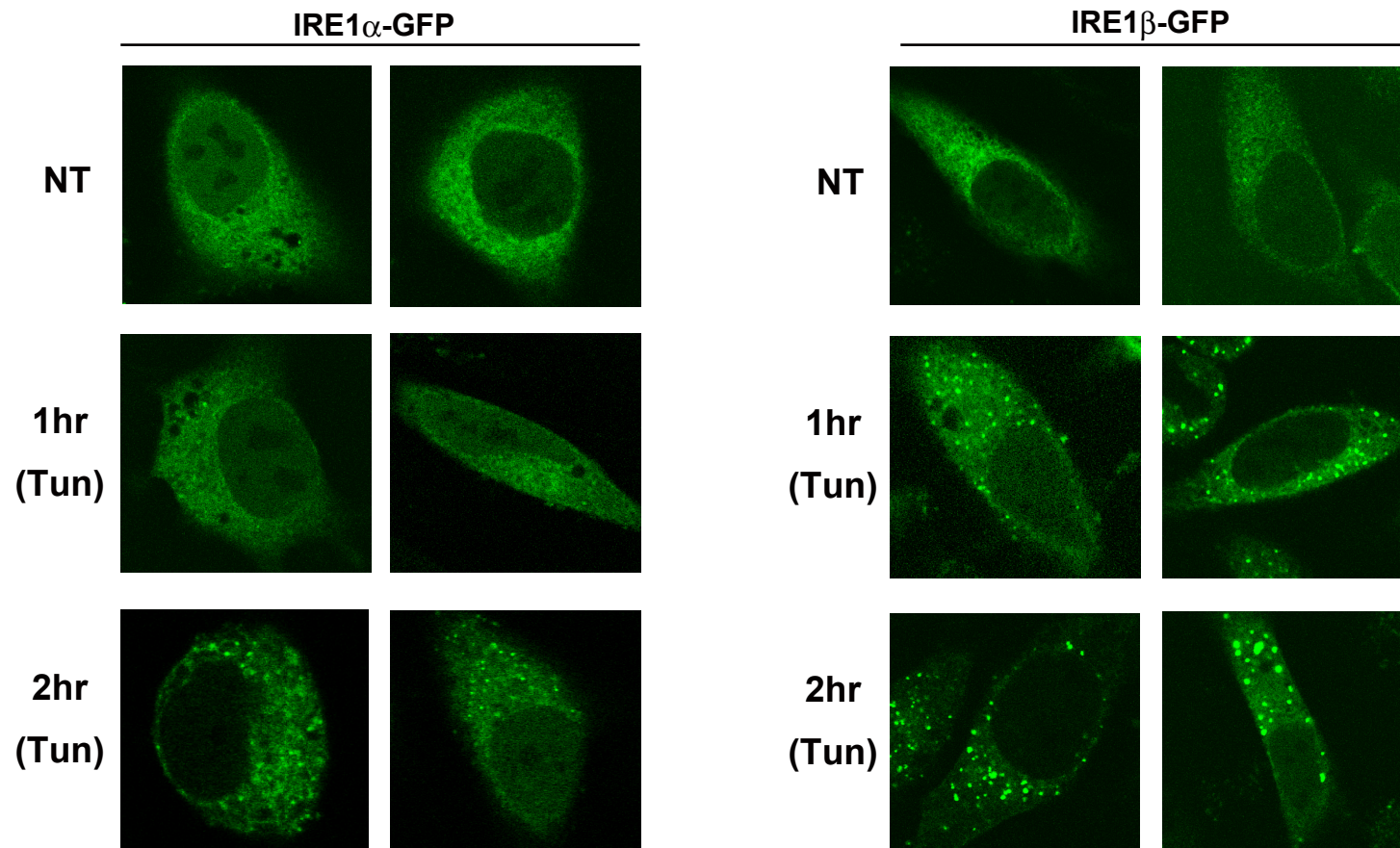

Supplement: Figure S2 — Timing of cluster formation of IRE1α and IRE1β upon tunicamycin treatment. GFP-fused IRE1s were transfected into HeLa cells, and treated with tunicamycin (2.5 µg/ml) for the indicated time. Fluorescent images were collected from untreated, 1-h treated, or 2-h treated cells. (PDF) [file pone.0051290.s002.pdf]

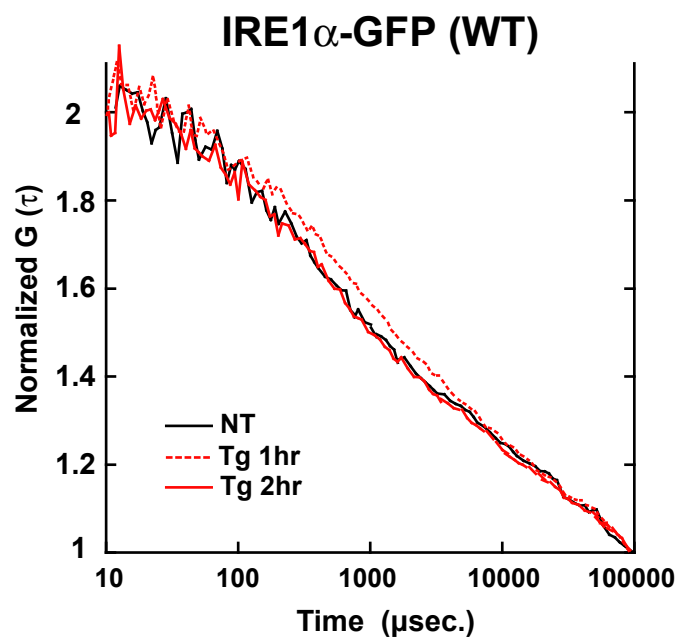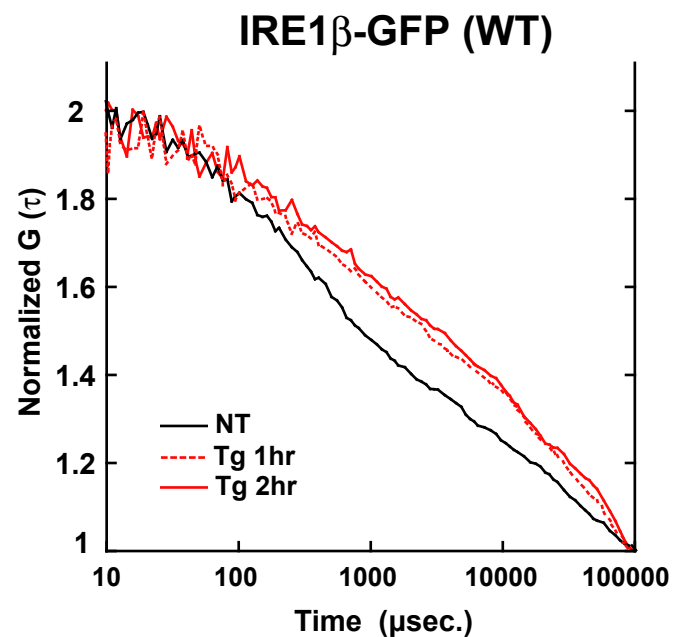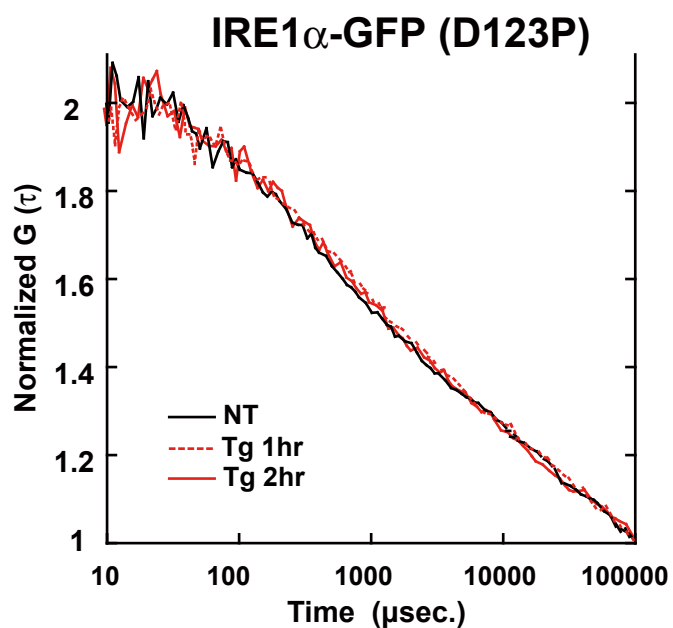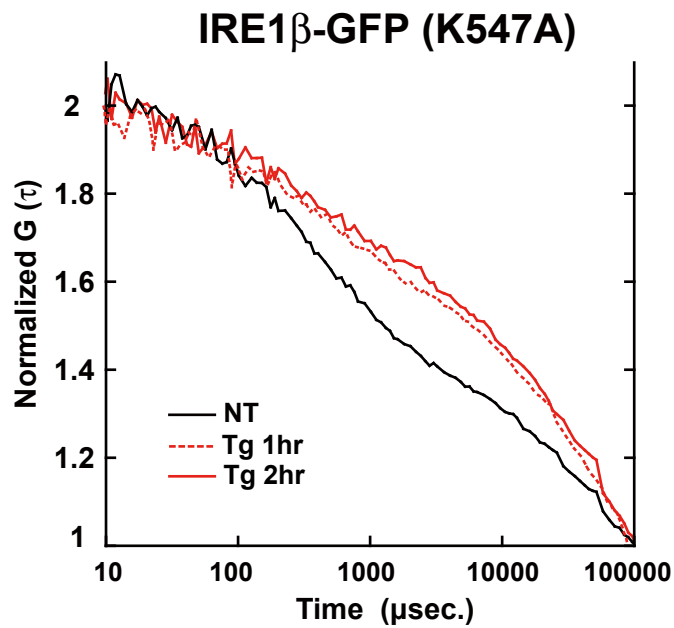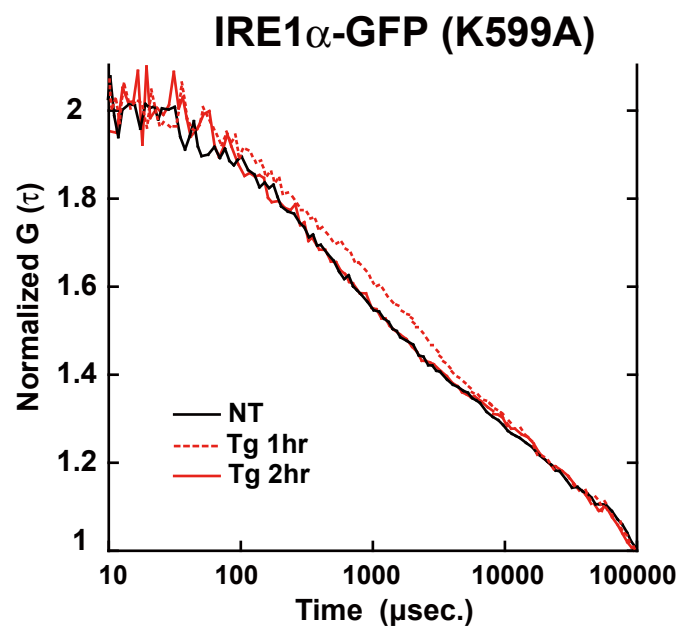

Supplement: Figure S3 — FCS analysis with mutants of IRE1α or IRE1β. GFP-fused IRE1s were transfected into HeLa cells and treated with or without thapsigargin (1 µM) for the indicated time, after which FCS measurement was performed. Normalized G(τ) was shown. (PDF) [file pone.0051290.s003.pdf]
